# Association between the incidence of infusion-related reactions by obinutuzumab and the dose of corticosteroid as premedication: a multicenter retrospective cohort study

**DOI:** 10.1186/s40780-026-00546-6

**Published:** 2026-02-03

**Authors:** Tatsuya Ohtsubo, Kazuhiro Yamamoto, Saori Matumoto, Kaori Ito, Yuzuka Sasa, Kosuke Tomishima, Satoshi Dote, Katsuya Makihara, Yoshinori Wakasugi, Tsutomu Mitsuie, Kouhei Yamagiwa, Kazuo Sato, Hiroki Hasegawa, Nobuhiko Uoshima, Yumi Kitahiro, Kanji Tomogane

**Affiliations:** 1 Department of Pharmacy, Japanese Red Cross Kyoto Daini Hospital, Kyoto, Japan; 2 Department of Integrated Clinical and Basic Pharmaceutical Sciences, Faculty of Medicine, Dentistry and Pharmaceutical Sciences, Okayama University, Okayama, Japan; 3 Department of Pharmacy, Kobe University Hospital, Kobe, Japan; 4 Department of Pharmacy, Japanese Red Cross Osaka Hospital, Osaka, Japan; 5 Faculty of Pharmacy, Meijo University, Nagoya, Japan; 6 Department of Pharmacotherapeutics and Informatics, Fujita Health University School of Medicine, Toyoake, Japan; 7 Department of Pharmacy, Kindai University Hospital, Sakai, Japan; 8 Department of Pharmacy, Japanese Red Cross Kyoto Daiichi Hospital, Kyoto, Japan; 9 Department of Pharmacy, Kyoto-Katsura Hospital, Kyoto, Japan; 10 Department of Pharmacy, Yodogawa Christian Hospital, Osaka, Japan; 11 Department of Pharmacy, Shiga University of Medical Science Hospital, Otsu, Japan; 12 Department of Pharmacy, Japanese Red Cross Otsu Hospital, Otsu, Japan; 13 Department of Pharmacy, Saiseikai Shiga Hospital, Ritto, Japan; 14 Department of Pharmacy, Japan Baptist Hospital, Kyoto, Japan; 15 Department of Pharmacy, Rakuwakai Otowa Hospital, Kyoto, Japan; 16 Department of Hematology, Japanese Red Cross Kyoto Daini Hospital, Kyoto, Japan

**Keywords:** Obinutuzumab, Infusion-related reaction, Premedication, Corticosteroids, Histamine 1 receptor antagonists

## Abstract

**Background:**

Premedication with corticosteroids is recommended for prophylaxis against infusion-related reactions (IRRs) caused by obinutuzumab despite a lack of solid evidence regarding the dose of corticosteroids.

**Methods:**

The incidence rates of IRR in the high-dose and low-dose corticosteroid groups were investigated and compared using Student’s t-test.Univariable and multivariable logistic regression analyses were performed on patients to explore the risk of developing IRRs with obinutuzumab.

**Results:**

The incidence of IRRs in the high-dose and low-dose corticosteroid groups at the initial administration of obinutuzumab was 27.0% (41/152) and 48.4% (31/64), respectively, indicating that the high-dose group had a lower incidence of IRRs (*p* = 0.002). The incidence of IRRs at the initial administration of obinutuzumab was significantly associated with the administration of first-generation histamine 1 receptor antagonist (OR = 3.31, 95% CI: 1.16–9.47; reference: second-generation histamine 1 receptor antagonist), hydrocortisone (OR = 7.21, 95% CI: 1.57–33.15; reference: dexamethasone), and methylprednisolone (OR = 3.99, 95% CI :1.13–14.10; reference: dexamethasone), although no association was found with the lower dose of corticosteroids.

**Conclusions:**

Although no association was found between corticosteroid dosage and IRR when considering multiple factors, dexamethasone may be a better option than hydrocortisone or methylprednisolone for preventing IRR. Additionally, second-generation H1-receptor antagonists may be a better option than first-generation drugs. Certain combinations of premedications may influence infusion reaction incidence.

**Supplementary Information:**

The online version contains supplementary material available at 10.1186/s40780-026-00546-6.

## Background

Obinutuzumab is a humanized anti-CD20 monoclonal antibody that is widely used for CD20-positive follicular lymphoma and chronic lymphocytic leukemia (including small lymphocytic lymphoma). In the GALLIUM study [1], which included patients with follicular lymphoma, patients who received obinutuzumab-based immunochemotherapy showed longer progression-free survival than those who received rituximab-based immunochemotherapy, making obinutuzumab-based immunochemotherapy a standard therapy for CD20-positive follicular lymphoma [2]. However, infusion-related reactions (IRRs) are observed as serious and frequent adverse events associated with obinutuzumab administration, which tend to occur during the initial administration [1, 3]. The incidence of IRRs in the obinutuzumab and rituximab groups in the GALLIUM study was 59% and 49% for all grades, respectively, and 11% and 5% for grades 3–5, respectively [1]. Obinutuzumab may result in the occurrence of IRRs more frequently than rituximab. Premedication with antipyretic analgesics, histamine 1 receptor antagonists, and corticosteroids is recommended for preventing IRRs caused by obinutuzumab [4, 5]. The U.S. Food and Drug Administration (FDA) and the European Medicines Agency (EMA) recommend corticosteroids such as prednisolone 100 mg, dexamethasone 20 mg, or methylprednisolone 80 mg, and explicitly state in the obinutuzumab package insert that hydrocortisone should not be used for IRR prevention because it is ineffective [4, 5]. However, the underlying evidence supporting this recommendation has not been established. Furthermore, although corticosteroid doses are expected to be reduced due to factors such as the presence of diabetes or the need to avoid severe infections, no published data exist regarding the corticosteroid doses, and no established dosing regimen is available. We aimed to standardize corticosteroid premedication at the time of initial obinutuzumab administration and to examine the association between corticosteroid dosage and IRR incidence.

## Methods

### Study participants

This multicenter retrospective cohort study was conducted at Japanese Red Cross Kyoto Daini Hospital, Fujita Health University Hospital, Japanese Red Cross Osaka Hospital, Kindai University Hospital, Japanese Red Cross Kyoto Daiichi Hospital, Kyoto Katsura Hospital, Yodogawa Christian Hospital, Shiga University of Medical Science Hospital, Japanese Red Cross Otsu Hospital, Saiseikai Shiga Hospital, Japan Baptist Hospital and Rakuwakai Otowa Hospital. Patients treated with obinutuzumab as initial therapy for primary or relapsed CD20-positive follicular lymphoma from August 1, 2018, to October 31, 2021, at collaborating institutions were included. The exclusion criteria were as follows: patients who take antipyretic analgesics (including nonsteroidal anti-inflammatory drugs), histamine 1 receptor antagonists, or corticosteroids other than those included in the regimen used before obinutuzumab administration and those who were not given corticosteroids as premedication on the same day as obinutuzumab administration.

### Definition of the high-dose and low-dose corticosteroid groups

The high-dose group comprised patients who received 100 mg or higher of prednisolone, 80 mg or higher of methylprednisolone, or 16.5 mg or higher of dexamethasone as premedication. The low-dose group comprised patients who received a dose lower than that in the high-dose group or those who took hydrocortisone. The cutoff dose value for the high-dose and low-dose groups was established based on recommendations from the FDA and EMA. However, for dexamethasone, since domestic formulations display the dexamethasone content without phosphate, the reference values for low-dose and high-dose dexamethasone in this study were set at 16.5 mg, which was considered equivalent to 20 mg of dexamethasone phosphate. The corticosteroid equivalent was set as 20 mg of hydrocortisone, 5 mg of prednisolone, 4 mg of methylprednisolone, and 0.75 mg of dexamethasone in equal doses [6].

### Definition of IRRs

IRRs incidence was determined from a physician’s documentation in the electronic medical record that IRRs (e.g., anaphylaxis, hypotension, nausea, chills, bronchospasm, pharyngeal or throat irritation, wheezing, laryngeal edema, atrial fibrillation, tachycardia, and hypersensitivity) occurred during obinutuzumab administration. In cases where there was no description regarding the incidence of IRRs in medical records, the attending physician performed a retrospective evaluation. The severity of IRRs was assessed by referring to the physician’s medical record. If no record was available regarding severity, each principal investigator assessed it based on “Infusion related reaction” in Common Terminology Criteria for Adverse Events version 5.0.

### Statistical analysis

Continuous variables are presented as means (standard deviations) and were compared using Student’s t-test. Nominal variables are presented as numbers and percentages and were compared using the chi-square test. Univariable and multivariable logistic regression analyses were performed on patients without missing values to explore the risk of developing IRRs with obinutuzumab. The explanatory variables were staging (AnnArbor classification), B symptoms, sIL-2R, LDH, dosage and type of corticosteroid, and type of histamine antagonist [7–9]. Statistical analyses were performed using Statistical Package for the Social Sciences (version 23.0; IBM Corp., New York) with a significance level of 5%.

## Results

### Patient characteristics

**Fig. 1:**
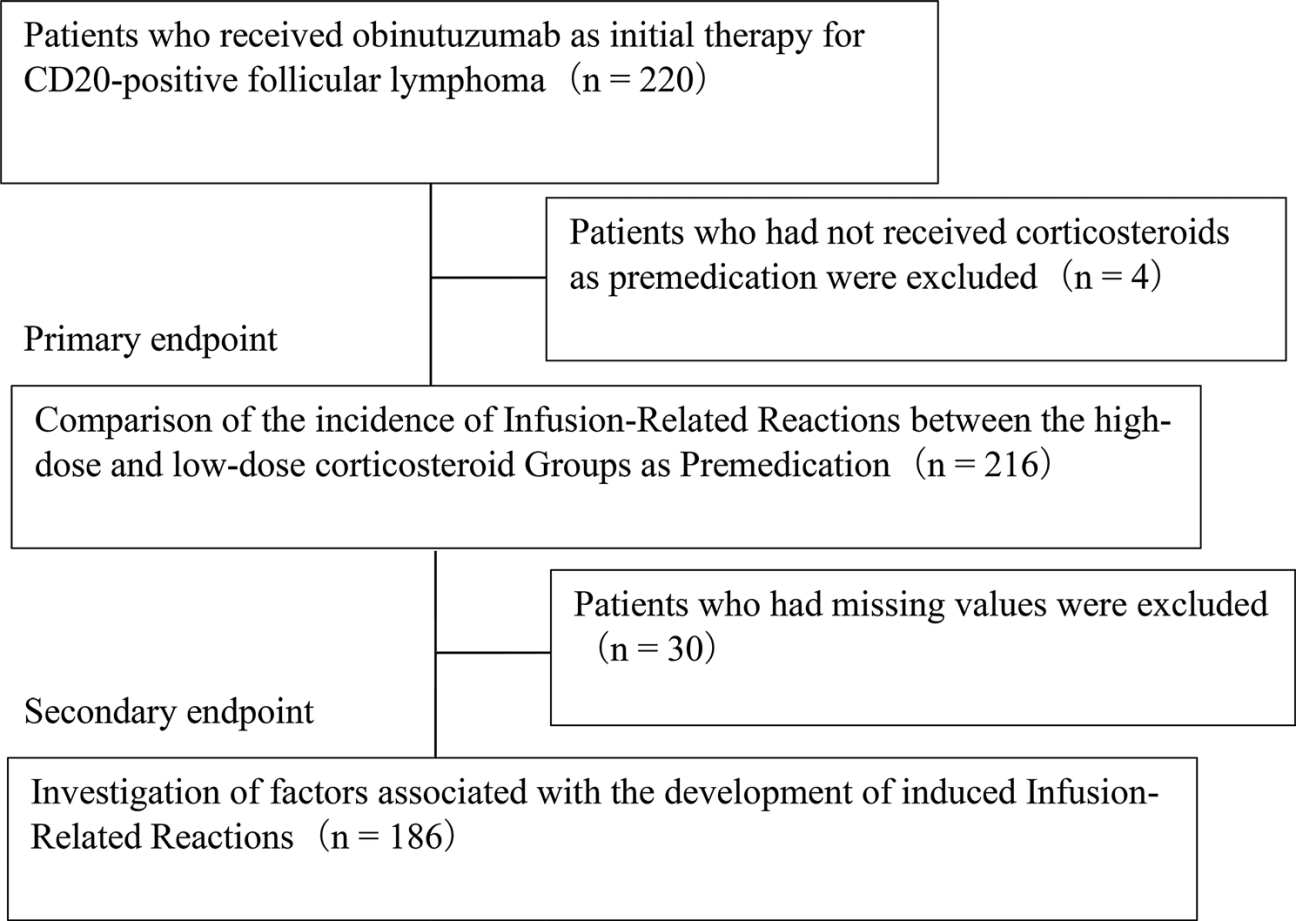
Research design overview

Of the 220 patients who received obinutuzumab as initial therapy for CD20-positive follicular lymphoma, 216 were included, excluding four patients who did not receive corticosteroids as premedication on the same day as obinutuzumab administration (Fig. 1). The patient backgrounds in the high- and low-dose corticosteroid groups are shown in Table 1. The number of patients in the high- and low-dose groups was 152 and 64, respectively. The high-dose group had a significantly lower age and a significantly lower percentage of patients with clinical stage III or higher than the low-dose group. Furthermore, the high-dose group had a significantly higher percentage of patients with PS of 0 and untreated patients, significantly higher ALP levels, and a higher percentage of patients with azole antifungal drug use. The details of corticosteroids, histamine 1 receptor antagonists, and antipyretic analgesics used as premedication are shown in Table 2. The most commonly used corticosteroid was dexamethasone [*n* = 151 (69.9%)]. The most commonly used histamine 1 receptor antagonist and antipyretic analgesic were d-chlorpheniramine maleate [*n* = 96 (44.4%)] and acetaminophen [*n* = 125 (57.9%)], respectively.

**Table 1:** Comparison of patient characteristics between high-dose and low-dose corticosteroid groups

		High dose (*n* = 152)	Low dose (*n* = 64)	*p*-value
Age [Mean (SD)]		66.4 (10.3)	69.9 (9.2)	0.019
Sex, [n (%)]	Male	77 (50.7)	35 (54.7)	0.59
PS [n (%)]	0	115 (75.6)	31 (48.4)	0.034
	≧ 1	36 (23.7)	20 (31.3)	
	Unknown	1 (0.7)	13 (20.3)	
Ann Arbor Staging [n (%)]	< Ⅲ	35 (23.0)	5 (7.8)	0.012
	≧Ⅲ	114 (75.0)	55 (85.9)	
	Unknown	3 (2.0)	4 (6.3)	
Line [n (%)]	Untreated	94 (61.8)	29 (45.3)	0.025
	Relapsed	58 (38.2)	35 (54.7)	
BM involvement [n (%)]	Yes	56 (36.8)	29 (45.3)	0.066
	No	90 (59.3)	26 (40.6)	
	Unknown	6 (3.9)	9 (14.1)	
Splenomegaly [n (%)]	Yes	32 (21.1)	12 (18.8)	0.67
	No	118 (77.6)	52 (81.2)	
	Unknown	2 (1.3)	0 (0)	
B symptoms [n (%)]	Yes	20 (13.2)	10 (15.6)	0.29
	No	129 (84.8)	41 (64.1)	
	Unknown	3 (2.0)	13 (20.3)	
ALP (U/L) (Mean, SD)		224.6 (150.8)	182.2 (91.1)	0.042
sIL-2R (U/mL) (Mean, SD)		1925.6 (2948.2)	2602.4 (4091.1)	0.17
LDH (U/L) (Mean, SD)		211.8 (74.5)	229.3 (105.2)	0.17
Hb (g/dL) (Mean, SD)		12.7 (1.8)	12.8 (2.1)	0.81
Combination regimen [n (%)]				
Bendamustine		127 (83.6)	58 (90.6)	0.282
CHOP		21 (13.8)	4 (6.3)	
CVP		4 (2.6)	2 (3.1)	
Concomitant drugs [n (%)]				
Aprepitant		58 (38.2)	21 (32.8)	0.46
Azole antifungal drugs		25 (16.4)	3 (4.7)	0.02
Macrolide antibiotics		1 (0.7)	0 (0)	0.52
Antipyretic analgesic				
Acetaminophen		85	40	0.37
NSAIDs		67	24	
Types of corticosteroids [n (%)]				
DEX		120 (79.0)	31 (48.4)	< 0.01
HDC		0 (0)	28 (43.8)	
PSL		11 (7.2)	3 (4.7)	
mPSL		21 (13.8)	2 (3.1)	
Types of histamine antagonists [n (%)]				
First generations		74 (48.7)	56 (87.5)	< 0.01
Second generations		67 (44.1)	8 (12.5)	
First and second concomitant		11 (7.2)	0 (0)	

PS: performance status, BM: bone marrow, ALP: alkaline phosphatase, sIL-2R: soluble interleukin-2 receptor, LDH: lactate dehydrogenase, Hb: hemoglobin, CHOP: cyclophosphamide, doxorubicin, vincristine, prednisolone, CVP: cyclophosphamide, vincristine, prednisolone, NSAIDs: non-steroidal anti-inflammatory drug, DEX: dexamethasone, HDC: hydrocortisone, PSL: prednisolone, mPSL: methylprednisolone

**Table 2:** Summary of premedications for administration of obinutuzumab

Category	Drug	Dose	Administration route	*n*
Corticosteroid	DEX	3.3 mg	i.v.	3
		6.6 mg	i.v.	26
		9.9 mg	i.v.	2
		16.5 mg	i.v.	32
		19 mg	i.v.	11
		19.8 mg	i.v.	68
		33 mg	i.v.	9
	HDC	15 mg	i.v.	1
		100 mg	i.v.	10
		200 mg	i.v.	16
		500 mg	i.v.	1
	PSL	30 mg	p.o.	2
		60 mg	p.o.	1
		100 mg 100 mg	p.o. i.v.	6 5
	mPSL	40 mg	i.v.	1
		62.5 mg	i.v.	1
		80 mg	i.v.	18
		100 mg	i.v.	1
		125 mg	i.v.	1
		250 mg	i.v.	1
Antipyretic analgesics	Acetaminophen	400 mg	p.o.	12
		500 mg	p.o.	57
		600 mg	p.o.	24
		800 mg	p.o.	3
		900 mg	p.o.	6
		1000 mg	p.o.	6
		1000 mg	i.v.	17
	Diclofenac	25 mg	p.o.	5
	Loxoprofen	60 mg	p.o.	86
Antihistamine	d-Chlorpheniramine maleate (First generation)	2 mg	p.o.	4
		4 mg	p.o.	1
		5 mg	i.v.	59
		6 mg	p.o.	30
		12 mg	p.o.	2
	Diphenhydramine hydrochloride (First generation)	30 mg	p.o.	25
		40 mg	p.o.	1
		50 mg	p.o.	8
	Epinastine (Second generation)	20 mg	p.o.	62
	Mequitadine (Second generation)	3 mg	p.o.	3
	Levocetirizine hydrochloride (Second generation)	2.5 mg	p.o.	1
		5 mg	p.o.	9
	Hydroxyzine pamoate (First generation) and levocetirizine hydrochloride (Second generation)	25 mg 5 mg	p.o.	11

DEX: dexamethasone, HDC: hydrocortisone, PSL: prednisolone, mPSL: methylprednisolone

### IRRs incidence according to the corticosteroid dose group and combinations of high-dose/low-dose corticosteroids and second-generation/first-generation antihistamines

The number of patients who had IRRs in the high-dose corticosteroid group was 41 (27.0%), which was significantly lower than that in the low-dose group [*n* = 31 (48.4%), *p* = 0.002] (Table 3). The severity of IRRs incidence in the high-dose group was grade 1 in 14 patients, grade 2 in 23 patients, and grade 3 in 4 patients, whereas, in the low-dose group, the severity of IRRs incidence was grade 1 in 8 patients, grade 2 in 20 patients, and grade 3 in 3 patients. Supplemental Table 1 shows the background of the 151 patients who received dexamethasone as premedication in the high- and low-dose corticosteroid groups. In the subgroup for the use of dexamethasone, the number of patients who had IRRs in the high- and low-dose groups was 26 (21.7%) and 10 (32.3%), respectively, with no significant difference (*p* = 0.22). The severity of IRRs in the high-dose group was grade 1 in 7 patients, grade 2 in 15 patients, and grade 3 in 4 patients. In the low-dose group, the severity of IRRs was grade 1 in 3 patients, grade 2 in 5 patients, and grade 3 in 2 patients. The number of patients who had IRRs in the High-dose corticosteroid/2nd generations histamine antagonists, High-dose corticosteroid/ 1st generations histamine antagonists, Low-dose corticosteroid/2nd generations histamine antagonists, and Low-dose corticosteroid/1st generations histamine antagonists was 11(14.7%), 27(40.3%), 2(50%), and 17(42.5%), respectively.

**Table 3:** Incidence of the infusion‑related reactions according to the dose of corticosteroid as premedication

IRRs (any grade), *n* (%)	High dose (*n* = 152)	Low dose (*n* = 64)	*p*-value
	41 (27.0)	31 (48.4)	0.002
Grade 0	111	33	-
Grade 1	14	8	-
Grade 2	23	20	-
Grade 3	4	3	-

IRRs: infusion‑related reactions

### Associated factors for the development of IRRs

Excluding 30 patients with missing values from 216 eligible patients, we explored associated factors for the incidence of IRRs in 186 patients (Fig. 1). Multivariable logistic regression analysis showed that first-generation histamine 1 receptor antagonist use as premedication [odds ratio (OR) = 3.31, 95% confidence interval (CI): 1.16–9.47; reference: second-generation histamine 1 receptor antagonist use], hydrocortisone use as premedication [OR = 7.21, 95% CI: 1.57–33.15; reference: dexamethasone], and methylprednisolone use as premedication [OR = 3.99, 95% CI: 1.13–14.10; reference: dexamethasone] were significantly associated with the incidence of IRRs during initial obinutuzumab administration (Table 4). Variance inflation factor (VIF) for the dosage and type of corticosteroids and the type of histamine antagonists was 1.048, 1.048, and 1.096, respectively. Low corticosteroid doses were not associated with the incidence of IRRs [OR = 0.84, 95% CI: 0.31–2.26] in the multivariable analysis. Prednisolone equivalent was not associated with the incidence of IRRs [OR = 0.99, 95% CI: 0.99–1.00] (Supplemental Table 2).

**Table 4:** Logistic regression analysis

Variables		Univariable analysis		Multivariable analysis	
	Category	OR (95% CI)	*p*-values	OR (95% CI)	*p*-values
AnnArbor classification	≥ Ⅲ	3.04 (1.20–7.73)	0.02	2.17 (0.75–6.30)	0.15
B symptoms	+	2.94 (1.31–6.59)	0.01	1.37 (0.52–3.60)	0.52
sIL-2R (U/mL)	-	1.0 (1.0–1.0)	0.35	1.0 (1.0–1.0)	0.16
LDH (U/L)	-	1.0 (0.99–1.0)	0.25	1.0 (0.99–1.0)	0.08
Types of Antipyretic analgesics	Acetaminophen	Reference	-	Reference	-
	NSAIDs			0.82 (0.31–2.11)	0.67
Dosage of corticosteroids	Low dose	2.10 (1.03–4.20)	0.04	0.84 (0.31–2.26)	0.73
Types of corticosteroids	DEX	Reference	-	Reference	-
	HDC	6.88 (2.19–21.57)	< 0.01	7.21 (1.57–33.15)	0.013
	PSL	4.01 (1.26–12.78)	0.02	2.52 (0.66–9.65)	0.18
	mPSL	3.44 (1.20–9.88)	0.02	3.99 (1.13–14.10)	0.032
Types of histamine antagonists	Second generations	Reference	-	Reference	-
	First generations	4.58 (2.06–10.19)	< 0.01	3.31 (1.16–9.47)	0.03
	First and second concomitant	3.75 (0.91–15.41)	0.07	2.38 (0.46–12.31)	0.30

sIL-2R: soluble interleukin-2 receptor, LDH: lactate dehydrogenase, NSAIDs: non-steroidal anti-inflammatory drug, DEX: dexamethasone, HDC: Hydrocortisone, PSL: prednisolone, mPSL: methylprednisolone

## Discussion

Patients who received high-dose corticosteroids prior to obinutuzumab administration had a lower incidence rate of IRR compared to the low-dose corticosteroid group. However, the corticosteroid dose itself was not recognized as an independent risk factor. On the other hand, hydrocortisone or methylprednisolone and first-generation H1 antihistamines were identified as factors potentially influencing the incidence of obinutuzumab-induced IRR. Furthermore, the combination of high-dose corticosteroids and second-generation antihistamines was suggested to be associated with a lower incidence of IRRs. This study is the first to report findings regarding the dosage and type of corticosteroids and the type of antihistamines in the prevention of obinutuzumab-induced IRR.

The use of high-dose corticosteroids may be an effective preventive measure against IRR. The high-dose group in this study was defined based on FDA recommendations, suggesting that the use of sufficient corticosteroids as recommended dose is important for preventing IRRs. Studies have suggested that corticosteroids have a dose-dependent effect on IRRs prophylaxis and that intravenously administered dexamethasone has a dose-dependent effect on preventing IRRs in the case of trastuzumab administration [10]. Although the report targeted patients who received trastuzumab therapy, this report supports the dose-dependent effect of corticosteroids on IRRs. Moreover, although some patient backgrounds significantly differed between the high- and low-dose groups in this study, they were not identified as risk factors for IRRs incidence in a previous study including patients treated with rituximab [7–9]; therefore, it is likely that those factors did not affect the incidence of IRRs. Although high tumor volume and bone marrow involvement may be risk factor for IRRs incidence in patients receiving obinutuzumab [11, 12], no significant differences in sIL-2R or LDH and bone marrow involvement were observed between the two groups in this study. In addition, because aprepitant is known to inhibit CYP3A4 and may increase corticosteroid exposure, we examined its potential influence on corticosteroid dose classification. Among the 21 patients in the low-dose group who received concomitant aprepitant, no patient was considered to meet the high-dose definition based on the predefined corticosteroid potency assessment (data not shown); therefore, the potential impact of aprepitant on high-dose/low-dose classification was minimal. We also examined the proportion of patients receiving concomitant aprepitant in both the high-dose and low-dose groups and found no significant difference between the two groups (Table 1), suggesting that corticosteroid exposure was not systematically biased by aprepitant use. Therefore, the comparison of infusion-related reaction incidence between dose groups was unlikely to have been confounded by this pharmacokinetic interaction. The concomitant use of azole antifungals has been reported to potentiate the effects of corticosteroids [13]. In this study, a higher percentage of patients in the high-dose group received concomitant azole antifungals; although increased corticosteroid exposure may have contributed to the suppression of IRRs, the possibility that the use of concomitant azole antifungals contributed to the lower incidence of IRRs in the high-dose group cannot be ruled out.

On the other hand, being in the low-dose group was not identified as a risk factor for IRR occurrence (Table 4). Furthermore, prednisolone equivalent dose was not associated with IRR incidence (Supplementary Table 2). In this study, the comparison group was classified based on the dose and type of corticosteroid administered. To exclude the influence of corticosteroid type, a post-hoc analysis was performed in the subgroup of patients receiving dexamethasone, which had the largest number of cases. The results showed no difference in IRR incidence between the high-dose and low-dose groups. However, due to insufficient sample size, it was impossible to replicate equivalent incidence rates between high- and low-dose groups in the entire study population and detect a difference with sufficient statistical power; further research is needed. Furthermore, prednisolone equivalents may not accurately reflect antibody titers [6], and equivalents based on standardized conversion factors may not be significant covariates for IRR. Additionally, since premedication involved the combined use of corticosteroids, antipyretic analgesics, and antihistamines, factors other than the corticosteroid dosage were considered to potentially influence the outcome.

The use of hydrocortisone and methylprednisolone was shown to be associated with a higher incidence of infusion-related reactions (IRRs) compared with the use of dexamethasone. Furthermore, variance inflation factor values were assessed in the multivariable logistic regression analysis, and no multicollinearity was detected, supporting the robustness of these findings. Notably, no data are available in the FDA or EMA package inserts to support the use of hydrocortisone for reducing IRRs. Importantly, hydrocortisone is commonly administered intravenously at doses of 100–200 mg for the management or prevention of allergic or infusion-related reactions. Higher doses are generally reserved for severe or refractory cases [14, 15]. In the present study, 96.4% of patients receiving hydrocortisone were classified into the low-dose group when doses were converted to prednisolone-equivalent values, and high-dose hydrocortisone administration was uncommon. Therefore, the higher incidence of IRRs observed in patients receiving hydrocortisone may reflect limited prophylactic effectiveness under typical dosing conditions rather than an intrinsic adverse effect of hydrocortisone itself. In contrast, 91.3% of patients who received methylprednisolone were classified into the high-dose group after dose conversion, suggesting that differences in the pharmacological and pharmacokinetic profiles of each corticosteroid, in addition to dose, may have influenced the incidence of IRRs. Although dexamethasone is known to have a longer biological half-life than hydrocortisone or methylprednisolone [6], the mechanisms underlying its apparent efficacy in preventing IRRs require further investigation.

Hydrocortisone and methylprednisolone were identified as factors contributing to IRR occurrence, whereas prednisolone was not. This finding may be attributable to the fact that 64.3% (9/14) of patients who received prednisolone as premedication were administered the oral formulation. In addition to cytokine-induced infusion reactions, hypersensitivity reactions of an allergic nature are also known to occur following rituximab administration [16], accounting for approximately 5–10% of post-dose reactions [17, 18]. Because these reactions present with clinical features similar to IRRs, it is difficult to distinguish them in routine practice, and the outcomes collected in this study may include a mixture of these phenotypes. Succinate esters are known inducers of hypersensitivity [19], and injectable formulations of hydrocortisone, prednisolone, and methylprednisolone are manufactured as succinate esters due to their poor water solubility. Therefore, the use of these injectable corticosteroids may have contributed to an increased frequency of hypersensitivity reactions. However, the exact proportion of hypersensitivity as a phenotype of IRRs in this study remains unknown and warrants further investigation.

The administration of first-generation histamine 1 receptor antagonists was shown to be a possible factor for the incidence of IRRs. No studies have examined the effect of histamine 1 receptor antagonist on obinutuzumab-induced IRRs. A study comparing the histamine 1 receptor antagonists fexofenadine and bepotastine in terms of efficacy as IRRs countermeasures during the initial administration of rituximab reported that bepotastine significantly suppressed the incidence of IRRs compared with fexofenadine [20], suggesting that differences in pharmacokinetics and other aspects of histamine 1 receptor antagonists affect the incidence of IRRs. Studies investigating the preventive effect of diphenhydramine and cetirizine on IRRs in patients who received rituximab and other anticancer therapies have demonstrated that cetirizine may be a viable alternative for diphenhydramine in preventing IRRs [21]. A randomized controlled trial is ongoing to evaluate the efficacy of bepotastine besilate compared with hydroxyzine pamoate on IRR [22]. The first-generation H1-receptor antagonist d-Chlorpheniramine maleate administered in this study exhibited dose variability within the range of 2 to 12 mg, and it cannot be ruled out that it may have been insufficiently effective in cases involving low doses and oral administration. First-generation histamine 1 receptor antagonists are contraindicated in patients with prostatic hypertrophy and glaucoma, and their central nervous system depressant effects cause side effects, such as drowsiness and dizziness. Although the reasons why second-generation histamine 1 receptor antagonists are more effective as IRRs countermeasures than first-generation histamine 1 receptor antagonists have not been examined, it was considered desirable to use second-generation histamine 1 receptor antagonists, which have less anticholinergic and central nervous system depressant effects than first-generation histamine 1 receptor antagonists, from the perspective of side effects.

Premedication with obinutuzumab involves concomitant use of corticosteroids, antipyretic analgesics, and antihistamines, so we examined the effects of these combinations. Analgesics/antipyretics were not identified as factors affecting IRR (Table 4). Therefore, we further investigated the IRR incidence rate based on combinations of corticosteroid dosage and antihistamine generation. The results suggested that the combination of high-dose corticosteroids and second-generation antihistamines may be associated with a lower incidence of IRRs (Table 5). However, these findings should be interpreted cautiously and should not be taken as evidence that corticosteroid dose alone is protective.

**Table 5:** Incidence of IRR based on corticosteroid dosage and combination with histamine antagonists

	IRR group (*n* = 57)	No IRR group (*n* = 129)	*p*-value
High-dose corticosteroid and 2nd generations histamine antagonists, *n* (%)	11 (14.7)	64 (85.3)	< 0.01
High-dose corticosteroid and 1st generations histamine antagonists, n (%)	27 (40.3)	40 (59.7)	-
Low-dose corticosteroid and 2nd generations histamine antagonists, n (%)	2 (50.0)	2 (50.0)	
Low-dose corticosteroid and 1st generations histamine antagonists, n (%)	17 ( 42.5)	23 (57.5)	

IRRs: infusion‑related reactions

Factors for the incidence of IRRs by rituximab have been reported to include high LDH and sIL-2R level, low hemoglobin level, presence of B symptoms and bone marrow infiltration, indolent lymphoma, and bulky disease [7–9]. High tumor volume may be risk factor for IRRs incidence in patients receiving obinutuzumab [11]. In this study, factors that could reflect tumor volume, such as staging, B symptoms, sIL-2R, and LDH, were examined but not identified as factors. The Groupe d’Etude des Lymphomes Folliculaires has developed criteria for predicting tumor volume in follicular lymphoma [23], which included B symptoms, but not sIL-2R or LDH. It was considered possible that the level of sIL-2R and LDH are unlikely to immediately reflect tumor volume because follicular lymphoma progresses slowly. We investigated the effect of the administration sequence of chemotherapy and obinutuzumab on IRR occurrence. The treatment schedule for chemotherapy combined with obinutuzumab is described as follows. Bendamustine is administered on days 1 and 2. CHOP therapy consists of cyclophosphamide, doxorubicin, and vincristine on day 1, followed by prednisolone/methylprednisolone on days 1–5. CVP therapy consists of cyclophosphamide and vincristine on day 1, with prednisolone/methylprednisolone administered on days 1–5. Obinutuzumab was administered before and after this combination chemotherapy. Since the administration sequence was considered a potential factor affecting infusion reaction occurrence, it was also investigated in this study; however, no effect was observed. (Data not shown)

This study has several limitations. This study was a retrospective survey, and collecting data using a uniform evaluation method for IRRs was impossible. In the GALLIUM study, the incidence of IRRs in the obinutuzumab and rituximab groups was 59% and 49%, respectively [1]. Other retrospective studies on IRRs caused by rituximab have reported IRRs incidence rates of 32.9% [7] and 37.9% [8] at the initial administration of rituximab. Two retrospective studies for IRRs caused by obinutuzumab have reported IRRs incidence rates of 36% [24] and 55.6% [12], at the time of initial obinutuzumab administration. The incidence of IRRs in this study was 33.3%, which is comparable to those reported in previous retrospective studies, although it differs from clinical trials in which precise evaluation can be performed. The possibility that IRRs may be underestimated in real-world data is an important limitation in this study. Furthermore, since the stepwise administration rate of obinutuzumab and the timing of premedication were not investigated, it cannot be ruled out that deviations from the recommended administration method may have influenced the occurrence of IRR.Logistic regression analysis was performed on 186 patients. The eight factors were selected as explanatory variables in the multivariable analysis with reference to clinical findings from previous reports. However, IRRs occurred in 57 patients, which may have reduced the reliability and robustness of the analysis. Bone marrow infiltration was not selected as an explanatory variable in the logistic regression analysis due to missing these examination data.

## Conclusions

Dexamethasone may be more effective than hydrocortisone or methylprednisolone, and second-generation H1-antihistamines may be more effective than first-generation agents in preventing IRR, whereas corticosteroid dose alone was not. Specific combinations of premedications may influence the incidence of infusion reactions, but further investigation is needed.

## Electronic Supplementary Material

Below is the link to the electronic supplementary material.

Supplementary Material 1

## Data Availability

The datasets generated during and/or analyzed during the current study are available from the first author on reasonable request.

## Abbreviations

IRRs: Infusion-related reactions
FDA: Food and Drug Administration
EMA: European Medicines Agency
VIF: Variance Inflation Factor

## References

[1] Marcus R, Davies A, Ando K, Klapper W, Opat S, Owen C, et al. Obinutuzumab for the first-line treatment of follicular lymphoma. N Engl J Med. 2017;377:1331–44. 28976863 10.1056/NEJMoa1614598

[2] National Comprehensive Cancer Network Inc. NCCN clinical practices in oncology; B-cell lymphomas-verssion2 [Internet]. 2024 [cited 2024 April 30]. Available from: https://www.nccn.org/professionals/physician_gls/pdf/b-cell.pdf.

[3] Cheson BD, Chua N, Mayer J, Dueck G, Trněný M, Bouabdallah K, et al. Overall survival benefit in patients with rituximab-refractory indolent non-Hodgkin lymphoma who received obinutuzumab plus Bendamustine induction and obinutuzumab maintenance in the GADOLIN study. J Clin Oncol. 2018;36:2259–66. 29584548 10.1200/JCO.2017.76.3656

[4] Food & Drug Administration. Drugs @FDA: FDA-Approved Drugs. obinutuzumab drug label. November, 2013. Available from: https://www.accessdata.fda.gov/drugsatfda_docs/label/2022/125486s034lbl.pdf. Date accessed: July 17, 2024.

[5] European Medical Agency. Drugs @EMA: EMA-Approved Drugs. obinutuzumab drug label. July, 2014. Available from: https://www.ema.europa.eu/en/documents/product-information/gazyvaro-epar-product-information_en.pdf. Date accessed: July 17, 2024.

[6] Liu D, Ahmet A, Ward L, Krishnamoorthy P, Mandelcorn ED, Leigh R, et al. A practical guide to the monitoring and management of the complications of systemic corticosteroid therapy. Allergy Asthma Clin Immunol. 2013;9:30. 23947590 10.1186/1710-1492-9-30 PMC3765115

[7] Hayama T, Miura K, Uchiike A, Nakagawa M, Tsutsumi D, Sakagami M, et al. A clinical prediction model for infusion-related reactions to rituximab in patients with B cell lymphomas. Int J Clin Pharm. 2017;39:380-5. 10.1007/s11096-017-0429-3 28144804

[8] Tachi T, Yasuda M, Usui K, Umeda M, Nagaya K, Osawa T, et al. Risk factors for developing infusion reaction after rituximab administration in patients with B-cell non-Hodgkin’s lymphoma. Pharmazie. 2015;70:674–7. 26601425

[9] Hong J, Kim JY, Ahn HK, Lee SM, Sym SJ, Park J, et al. Bone marrow involvement is predictive of infusion related reaction during rituximab administration in patients with B cell lymphoma. Support Care Cancer. 2013;21:1145–52. 23111943 10.1007/s00520-012-1639-9

[10] Goto E, Hata T, Nishihara M, Neo M, Iwamoto M, Kimura K, et al. Preventive effect of dexamethasone premedication on the development of infusion-related reaction in breast cancer patients receiving trastuzumab. Br J Clin Pharmacol. 2023;89:2102–12. 36709967 10.1111/bcp.15675

[11] Freeman CL, Morschhauser F, Sehn L, Dixon M, Houghton R, Lamy T, et al. Cytokine release in patients with CLL treated with obinutuzumab and possible relationship with infusion-related reactions. Blood. 2015;126:2646–9. 26447188 10.1182/blood-2015-09-670802 PMC4671111

[12] Masamoto Y, Taoka K, Maki H, Kurokawa M. Bone marrow involvement is a risk factor for infusion-realated reactions in patients with follicular lymphoma treated by obinutuzumab,Annals of hematology. Ann Hematol. 2022;101:2795–7. 36192661 10.1007/s00277-022-04987-5

[13] Varis T, Kivistö KT, Backman JT, Neuvonen PJ. The cytochrome P450 3A4 inhibitor Itraconazole markedly increases the plasma concentrations of dexamethasone and enhances its adrenal-suppressant effect. Clin Pharmacol Ther. 2000;68:487–94. 11103751 10.1067/mcp.2000.110772

[14] Post TW, editor. Glucocorticoid therapy: Pharmacology and principles of use. In: UpToDate, Waltham. MA: UpToDate Inc.; Available at: https://www.uptodate.com. Accessed [January 8, 2026].

[15] Anaphylaxis MA. UpToDate Inc. Available at: https://www.uptodate.com. Accessed [January 8, 2026].

[16] Fouda GE, Bavbek S. Rituximab hypersensitivity; from clinical presentation to management. Front Pharmacol. 2020;11:572863. 33013416 10.3389/fphar.2020.572863 PMC7508176

[17] Brennan PJ, Rodriguez Bouza TR, Hsu FI, Sloane DE, Castells MC. Hypersensitivity reactions to mAbs: 105 desensitizations in 23 patients, from evaluation to treatment. J Allergy Clin Immunol. 2009;124:1259–66. 19910036 10.1016/j.jaci.2009.09.009

[18] Galvão VR, Castells MC. Hypersensitivity to biological agents—Updated diagnosis, management, and treatment. J Allergy Clin Immunol Pract. 2015;3:175–85. 25754718 10.1016/j.jaip.2014.12.006

[19] Kamm GL, Hagmeyer KO. Allergic-type reactions to corticosteroids. Ann Pharmacother. 1999;33:451–60. 10332537 10.1345/aph.18276

[20] Matsui N, Ishihara N, Mochizuki Y, Tamaki H, Nishimura N, Naora K. Inhibitory effect on rituximab-induced infusion reaction by premedication with Fexofenadine or bepotastine. Jpn J Pharm Health Care Sci. 2019;45:396–403.

[21] Durham CG, Thotakura D, Sager L, Foster J, Herrington JD. Cetirizine versus diphenhydramine in the prevention of chemotherapy-related hypersensitivity reactions. J Oncol Pharm Pract. 2019;25:1396–401. 30419768 10.1177/1078155218811505

[22] Kitahiro Y, Yamamoto K, Yakushijin K, Ioroi T, Tanda M, Itohara K, et al. The efficacy of bepotastine besilate compared with hydroxyzine pamoate for preventing infusion reactions to the first dose of rituximab in patients with Non-Hodgkin lymphoma: protocol for a phase II, Double-Blind, multicenter randomized trial. JMIR Res Protoc. 2024;13:e54882. 38386393 10.2196/54882 PMC10921330

[23] Brice P, Bastion Y, Lepage E, Brousse N, Haïoun C, Moreau P, et al. Comparison in low-tumor-burden follicular lymphomas between an initial no-treatment policy, prednimustine, or interferon alfa: A randomized study from the groupe d’etude des lymphomes Folliculaires. groupe d’etude des lymphomes de l’adulte. J Clin Oncol. 1997;15:1110–7. 9060552 10.1200/JCO.1997.15.3.1110

[24] Kuromatsu M, Kajita T, Taruno M, Nishikawa Y, Akasaka T, Okuno T. Search for risk factors influencing the occurrence of infusion reaction after initial treatment with obinutuzumab. Jpn J Pharm Health Care Sci. 2021;47:631–8.

