# Correction: Association between the incidence of infusion-related reactions by obinutuzumab and the dose of corticosteroid as premedication: a multicenter retrospective cohort study

**DOI:** 10.1186/s40780-026-00605-y

**Published:** 2026-07-17

**Authors:** Tatsuya Ohtsubo, Kazuhiro Yamamoto, Saori Matumoto, Kaori Ito, Yuzuka Sasa, Kosuke Tomishima, Satoshi Dote, Katsuya Makihara, Yoshinori Wakasugi, Tsutomu Mitsuie, Kouhei Yamagiwa, Kazuo Sato, Hiroki Hasegawa, Nobuhiko Uoshima, Yumi Kitahiro, Kanji Tomogane

**Affiliations:** 1 Department of Pharmacy, Japanese Red Cross Kyoto Daini Hospital, Kyoto, Japan; 2 Department of Integrated Clinical and Basic Pharmaceutical Sciences, Faculty of Medicine, Dentistry and Pharmaceutical Sciences, Okayama University, Okayama, Japan; 3 Department of Pharmacy, Kobe University Hospital, Kobe, Japan; 4 Department of Pharmacy, Japanese Red Cross Osaka Hospital, Osaka, Japan; 5 Faculty of Pharmacy, Meijo University, Nagoya, Japan; 6 Department of Pharmacotherapeutics and Informatics, Fujita Health University School of Medicine, Toyoake, Japan; 7 Department of Pharmacy, Kindai University Hospital, Sakai, Japan; 8 Department of Pharmacy, Japanese Red Cross Kyoto Daiichi Hospital, Kyoto, Japan; 9 Department of Pharmacy, Kyoto-Katsura Hospital, Kyoto, Japan; 10 Department of Pharmacy, Yodogawa Christian Hospital, Osaka, Japan; 11 Department of Pharmacy, Shiga University of Medical Science Hospital, Otsu, Japan; 12 Department of Pharmacy, Japanese Red Cross Otsu Hospital, Otsu, Japan; 13 Department of Pharmacy, Saiseikai Shiga Hospital, Ritto, Japan; 14 Department of Pharmacy, Japan Baptist Hospital, Kyoto, Japan; 15 Department of Pharmacy, Rakuwakai Otowa Hospital, Kyoto, Japan; 16 Department of Hematology, Japanese Red Cross Kyoto Daini Hospital, Kyoto, Japan

**Correction: J Pharm Health Care Sci (2026) 12:27**

10.1186/s40780-026-00546-6

In this article the author’s name Katsuya Makihara was incorrectly written as Katuya Makihara.

The original article has been corrected.

