# Supplementary material for: Association between the incidence of infusion-related reactions by obinutuzumab and the dose of corticosteroid as premedication: a multicenter retrospective cohort study
Source: J Pharm Health Care Sci. 2026 Feb 3;12:27. doi: 10.1186/s40780-026-00546-6 (PMC12958665; doi:10.1186/s40780-026-00546-6)
Supplement: Supplementary file 1 — Supplementary Material 1 [file 40780_2026_546_MOESM1_ESM.docx]

**Supplemental Table 1 Comparison of patient characteristics between high-dose and low-dose groups in patients using dexamethasone**

|  |  | High dose (n = 120) | Low dose (n = 31) | *p*-value |
| --- | --- | --- | --- | --- |
| Age [Mean (SD)] |  | 66.4 (10.0) | 70.5 (8.1) | 0.037 |
| Sex [n (%)] | Male | 62 (51.7) | 19 (61.2) | 0.34 |
| PS [n (%)] | 0 | 91 (75.8) | 14 (45.2) | 0.024 |
|  | ≧ 1 | 29 (24.2) | 12 (38.7) |  |
|  | Unknown | 0 (0) | 5 (16.1) |  |
| Ann Arbor Staging [n (%)] | < Ⅲ | 30 (25.0) | 2 (6.5) | 0.024 |
|  | ≧Ⅲ | 90 (75.0) | 29 (93.5) |  |
|  | Unknown | 0 (0) | 0 (0) |  |
| Line [n (%)] | Untreated | 73 (60.8) | 17 (54.8) | 0.54 |
|  | Relapsed | 47 (39.2) | 14 (45.2) |  |
| BM involvement [n (%)] | Yes | 42 (35.0) | 17 (54.8) | 0.026 |
|  | No | 75 (62.5) | 12 (38.7) |  |
|  | Unknown | 3 (2.5) | 2 (6.5) |  |
| Splenomegaly [n (%)] | Yes | 19 (15.8) | 7 (22.6) | 0.40 |
|  | No | 99 (82.5) | 24 (77.4) |  |
|  | Unknown | 2 (1.7) | 0 (0) |  |
| B symptoms [n (%)] | Yes | 10 (8.3) | 3 (9.7) | 0.62 |
|  | No | 108 (90.0) | 23 (74.2) |  |
|  | Unknown | 2 (1.7) | 5 (16.1) |  |
| ALP (U/L) (Mean, SD) |  | 211.4 (91.9) | 180.6 (73.8) | 0.087 |
| sIL-2R (U/mL) (Mean, SD) |  | 1925.2 (3205.0) | 2678.9 (2926.1) | 0.24 |
| LDH (U/L) (Mean, SD) |  | 205.6 (57.2) | 221.3 (107.6) | 0.27 |
| Hb (g/dL) (Mean, SD) |  | 13.0 (1.6) | 12.6 (2.5) | 0.32 |
| Combination regimen [n (%)] |  |  |  |  |
| Bendamustine |  | 113 (94.2) | 29 (93.5) | 0.84 |
| CHOP |  | 6 (5.0) | 2 (6.5) |  |
| CVP |  | 1 (0.8) | 0 (0) |  |
| Concomitant drugs [n (%)] |  |  |  |  |
| Aprepitant | Yes | 56 (46.7) | 6 (19.4) | < 0.01 |
|  | No | 64 (53.3) | 25 (80.6) |  |
| Azole antifungal drugs | Yes | 15 (12.5) | 3 (9.7) | 0.67 |
|  | No | 105 (87.5) | 28 (90.3) |  |
| Macrolide antibiotics | Yes | 1 (0.8) | 0 (0) | 0.61 |
|  | No | 119 (99.2) | 31 (100) |  |
| Types of histamine antagonists [n (%)] |  |  |  |  |
| 1st generations |  | 53 (44.2) | 31 (100) | < 0.01 |
| 2nd generations |  | 61 (50.8) | 0 (0) |  |
| 1st and 2nd concomitant |  | 6 (5.0) | 0 (0) |  |

PS, performance status; BM, bone marrow; ALP, alkaline phosphatase; sIL-2R, soluble interleukin-2 receptor; LDH, lactate dehydrogenase; Hb, hemoglobin; CHOP, cyclophosphamide, doxorubicin, vincristine, prednisolone; CVP, cyclophosphamide, vincristine, prednisolone

**Supplementarl Table 2** **Logistic regression analysis**

|  |  | Univariable analysis | |  | Multivariable analysis | |
| --- | --- | --- | --- | --- | --- | --- |
| Variables | Category | OR (95% CI) | *P*-values |  | OR (95% CI) | *P*-values |
| AnnArbor classification | ≥ Ⅲ | 3.04 (1.20–7.73) | 0.02 |  | 2.49 (0.95–6.56) | 0.064 |
| B symptoms | + | 2.94 (1.31–6.59) | 0.01 |  | 2.48 (1.06–5.80) | 0.037 |
| sIL-2R (U/mL) | - | 1.00 (1.0–1.0) | 0.35 |  | 1.00 (1.00–1.00) | 0.59 |
| LDH (U/L) | - | 1.00 (0.99-1.0) | 0.25 |  | 1.00 (0.99–1.00) | 0.10 |
| Dosage of corticosteroids  (Prednisolone equivalent) | - | 0.99 (0.99-1.00) | 0.065 |  | 0.99 (0.99–1.00) | 0.15 |

sIL-2R: soluble interleukin-2 receptor, LDH: lactate dehydrogenase
